# Temozolomide resistance in glioblastoma cells occurs partly through epidermal growth factor receptor-mediated induction of connexin 43

**DOI:** 10.1038/cddis.2014.111

**Published:** 2014-03-27

**Authors:** J L Munoz, V Rodriguez-Cruz, S J Greco, S H Ramkissoon, K L Ligon, P Rameshwar

**Affiliations:** 1 Rutgers-Graduate School of Biomedical Science, Newark, NJ, USA; 2 Department of Medicine, Hematology/Oncology, New Jersey Medical School, Rutgers School of Biomedical Health Sciences, Newark, NJ, USA; 3 Department of Pathology, Brigham and Women's Hospital, Boston Children's Hospital and Harvard Medical School, Boston, MA, USA; 4 Department of Medical Oncology, Dana-Farber Cancer Institute, Boston, MA, USA

**Keywords:** connexin 43, glioblastoma, resistance, temozolomide EGF receptor, stem cell, immune therapy

## Abstract

Glioblastoma Multiforme (GBM) is an aggressive adult primary brain tumor with poor prognosis. GBM patients develop resistance to the frontline chemotherapy, temozolomide (TMZ). As the connexins (Cx) have been shown to have a complex role in GBM, we investigated the role of Cx43 in TMZ resistance. Cx43 was increased in the TMZ-resistant low passage and cell lines. This correlated with the data in The Cancer Genome Atlas. *Cx43* knockdown, reporter gene assays, chromatin immunoprecipitation assay, real-time PCR and western blots verified a role for Cx43 in TMZ resistance. This occurred by TMZ-resistant GBM cells being able to activate epidermal growth factor receptor (EGFR). In turn, EGFR activated the JNK-ERK1/2-AP-1 axis to induce *Cx43*. The increased Cx43 was functional as indicated by gap junctional intercellular communication among the resistant GBM cells. Cell therapy could be a potential method to deliver drugs, such as anti-EGF to tumor cells. Similar strategies could be used to reverse the expression of Cx43 to sensitize GBM cells to TMZ. The studies showed the potential for targeting EGF in immune therapy. These agents can be used in conjunction with stem cell therapy to treat GBM.

Glioblastoma Multiforme (GBM), the most common adult primary brain tumor, has poor prognosis with <3% survival after 5 years of diagnosis.^1^ Currently, anti-neoplastic treatment combines chemotherapy, temozolomide (TMZ), radiotherapy and resectional surgery.^2^ TMZ is an alkylating agent that induces apoptosis through DNA strand breaks is considered as the frontline chemotherapeutic agent for GBM.^3^ Despite its frontline status, GBM patients commonly exhibit resistance to TMZ treatment.^4^ Although there are several reports aimed at understanding how GBM cells resist TMZ, the mechanism(s) remain unclear. The reports on chemoresistance include mismatch repair of genes, cell cycle alterations and the expression of ATP-dependent drug efflux pumps.^5^

A number of intracellular pathways have been described in the growth and survival of GBM. These include the activation of epidermal growth factor receptor (EGFR) in GBM cells. EGFR dysfunction correlates with poor prognosis.^6, 7^ GBM patients with increased *EGFR* expression have enhanced activation of the EGFR variant, EGFRvIII.^8^ The EGFR is a member of the Erb family of receptor tyrosine kinases. Activated EGFR is a homodimeric transmembrane protein that undergoes auto-transphosphorylation through intrinsic intracellular protein-tyrosine kinase domains.^9^ The activation of EGFR leads to downstream signaling pathways such as phosphorylation of AKT, MAPK and JNK.^10^

Chemoresistance of GBM cells can occur by intercellular communication through gap junction (GJIC).^11^ GJIC is established by the interaction between two hemichannels on adjacent cell membranes.^12^ The hemichannels are formed by members of the connexin (Cx) protein family. GJIC facilitates the exchange of small molecules and microRNA.^13, 14^ Cx43 can exert both tumor suppressor and oncogenic functions.^15^ Astrocytes and astrocytomas express high levels of Cx43.^16^ During epithelial-to-mesenchymal transition and the period of overt tumor infiltration, there is a loss of GJIC between the malignant cells.^17^ However, the role of Cx43 in the migration of GBM cells is mixed. While the literature reported on a requirement for decreased Cx43 for GBM cells to migrate to other sites,^17^ another study showed a facilitating role for Cx43 in GBM migration.^18^ Together, these two opposing reports underscored the complex role of Cx43 in the development of GBM. This study investigated the role of GJIC in the early phase of acquired chemoresistance.

Here we show an increase in Cx43 in TMZ-resistant GBM cells that formed GJIC between the resistant cells. This suggested that GJIC could be a method by which resistance is communicated among GBM cells. We demonstrated that *Cx43* expression was regulated at the level of transcription in the chemoresistant GBM cells. This occurred partly through the activation of EGFR1 that activated AP-1 to increase *Cx43* transcription.

## Results

### *Cx43* expression in TMZ-resistant GBM cells

**Figure 1:**
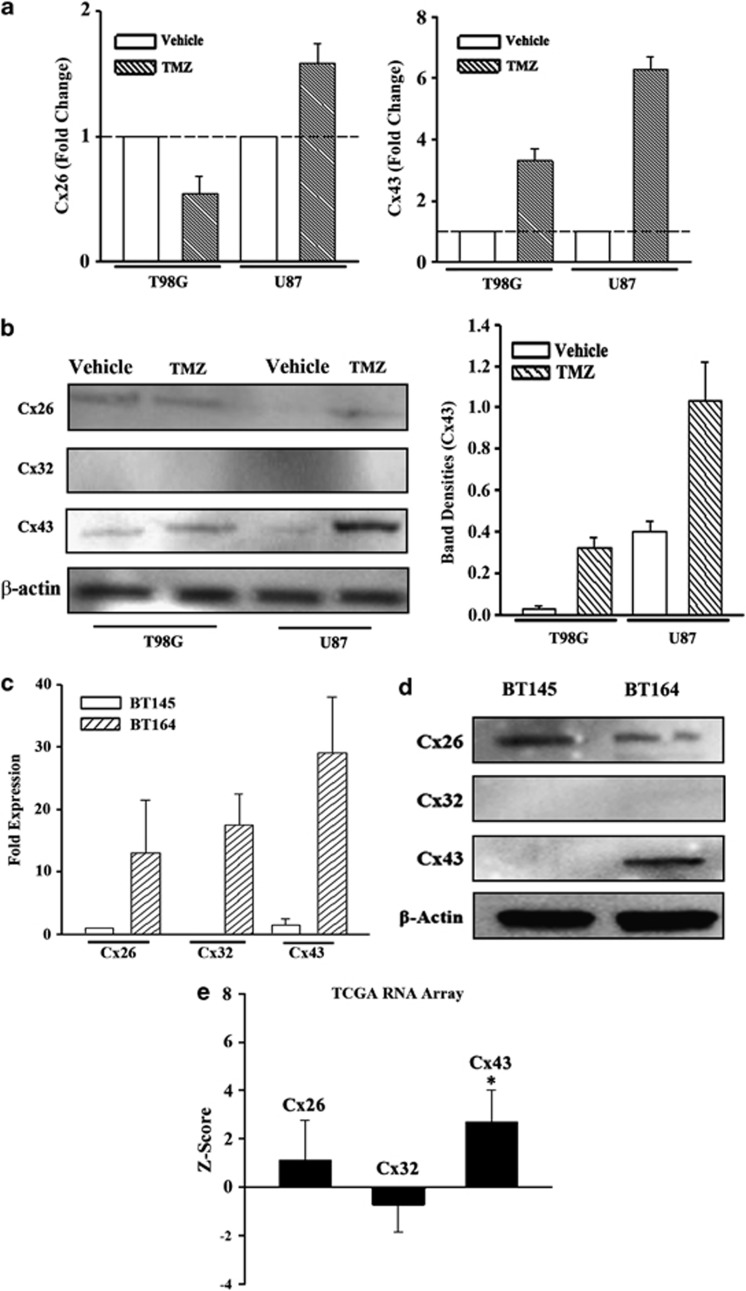
Increased Cx43 in TMZ-treated GBM cells. (**a**) U87 and T98G cells were treated with 200 *μ*M TMZ. After 72 h, real-time PCR was performed with primers specific for Cx26 (left) and Cx43 (right). (**b**) Whole-cell extracts were prepared from U87 and T98G cells treated with TMZ for 72 h, and then analyzed by western blots for Cx26, Cx32 and Cx43 (left). The normalized band densities for Cx43 protein is shown at right. (**c**) RNA from low-passage cell lines from a patient with recurrent GBM (TMZ resistance BT164) and a naive patient (TMZ-sensitive BT145) were studied by real-time PCR for Cx26, Cx32 and Cx43 mRNA. The data are shown for the mean relative fold expression±S.D., *n*=4. (**d**) Whole-cell extracts from BT145 and BT164 were analyzed by western blot for Cx43. (**e**) TCGA level 3 with >500 GBM tissues was used to verify the expressions of *Cx26, Cx32* and *Cx43* expressions. The results are shown as the mean±S.D. for all samples within the database.* *P*<0.05 *versus* Cx26 or Cx32

**Figure 2:**
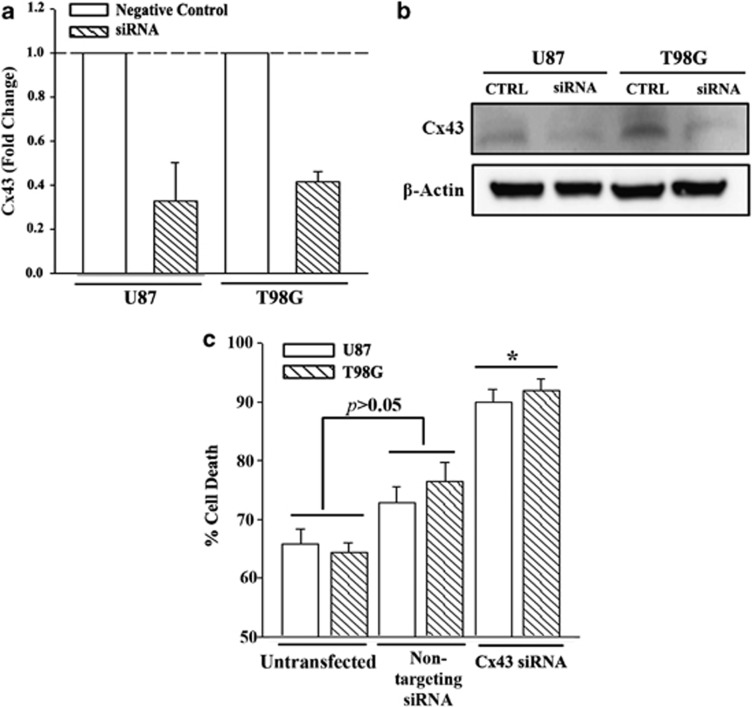
Cell viability of TMZ-treated *Cx43* knockdown GBM cells. U87 and T98G were transfected with Cx43-targeted siRNA or a non-targeting siRNA (Negative Control, Neg Ctrl). The cells were analyzed by real-time PCR for Cx43 mRNA (**a**) and whole-cell extracts in western blot with anti-Cx43 (**b**). The knockdown cells, Neg Ctrl and untransfected cells were treated with 200 *μ*M TMZ. After 72 h, the cells were studied for viability using the CytoTox 96 LDH release. The results are presented as mean±S.D. of triplicates from each of four independent experiments (*n*=12).*P<0.05 *versus* Neg Ctrl and untransfected cells

The role of GJIC has been extensively studied in the central nervous system.^19^ We asked whether TMZ resistance could be ‘transmitted' to other cells through GJIC. We first identified the Cx type on TMZ-resistant U87 and T98G cells. Chemoresistance was established with 200 *μ*M TMZ for 72 h.^20^ The viable cells were studied for *Cx* expression by real-time PCR and western blot. We selected *Cx26, Cx32* and *Cx43* because of their expression on neurons, Schwann cells and astrocytes, respectively.^19^ Cx43 mRNA was increased by four- to six—fold and its protein was increased by three- to four-fold; Cx26 mRNA was minimally increased and Cx32 mRNA was undetectable (CT>35) (Figures 1a and b). Similar findings were noted for the corresponding proteins (Figure 2b). In summary, *Cx43* expression was significantly increased in the TMZ-resistant GBM cells.

As commercial U87 and T98G were passaged for many generations, we asked whether *Cx* was similarly expressed in primary GBM cells, low-passage GBM cells from patients with naive (BT145) and recurring (resistant) (BT164) GBM. Real-time PCR for Cx26, Cx32 and Cx43 cDNA indicated significant (*P*<0.05) increases in BT164 (resistant) as compared with BT145 (naive) (Figure 1c). Western blot indicated a significant increase for Cx43 in the resistant BT164 cells but reduced Cx26 and undetectable Cx32 (Figure 1d).

We next analyzed the data in The Cancer Genome Atlas (TCGA) to determine whether Cx43 is increased in a large number of GBM tissues. The TCGA project has data analyzed using Agilent Gene Chip with >500 primary GBM tissues. Comparative analyses for *Cx43* expression were performed with the 522 available gene chip data, showed *Cx43* expression with a *Z*-score of 2.7 and a S.D. of 1.3. This indicated an average of 2.7-fold increase in *Cx43* expression in the GBM samples as compared with control gene expression. Similar analyses for Cx26 and Cx32 showed no significant (*P*>0.05) change as compared with control (Figure 1e). Taken together, TMZ-resistant GBM cells, including the low-passaged cell lines, combined with the data in the TCGA gene array database, which indicated that *Cx43* was increased in GBM.

### Sensitization of *Cx43* knockdown GBM cells to TMZ

The studies supported a molecular role for Cx43 in TMZ resistance GBM cells (Figure 1). Functional studies investigated the sensitivity of *GJA1* (*Cx43*) knockdown GBM cells. The specificity was studied with non-targeted siRNA (negative control). Real-time PCR and western blot verified the efficiency of *Cx43* knockdown (Figures 2a and b, Supplementary Figure S1). GBM cells were established as resistance to TMZ by treating with 200 *μ*M TMZ. At 72 h, the cells were analyzed with a LHD release assay for cell death. As expected, only the resistant cells survived TMZ treatment in the untransfected cells, shown as ∼35% (Figure 2c, untransfected). There was no significant difference (*P*>0.05) with a non-targeting siRNA. In contrast, transfection with Cx43 siRNA caused a significant (*P*<0.05) increase in cell death as compared with cells transfected with non-targeting siRNA (Figure 2c). These findings indicated that *Cx43* was involved in the acquired resistance of TMZ-treated GBM cells.

### TMZ activated the 5′ regulatory region of *GJA1* (*Cx43*) in GBM cells

**Figure 3:**
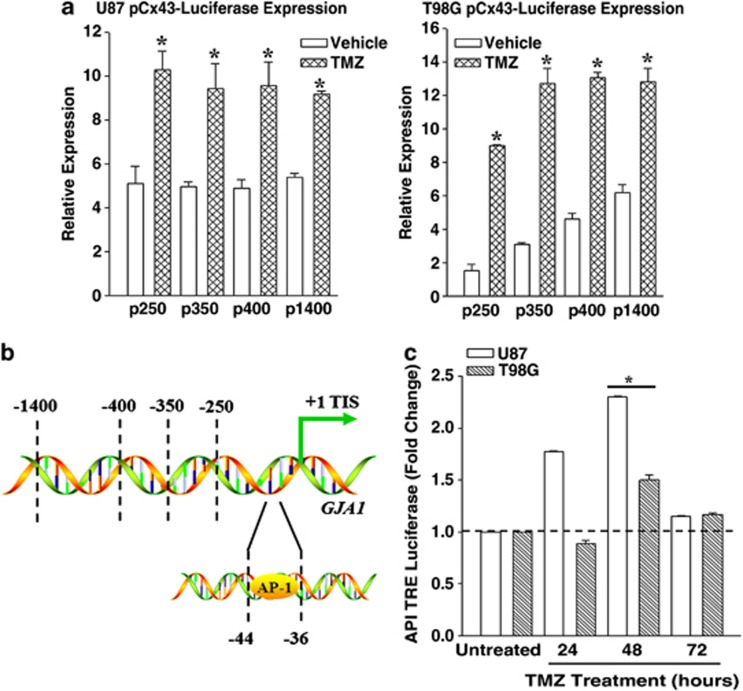
Effect of TMZ on the 5′ regulatory region of *GJA1* (*Cx43*). T98G and U87 cells were transfected with luciferase reporter vectors with various inserts, upstream of the transcriptional initiation site of the *GJA1* 5′ regulatory region.^27^ The transfectants were treated with vehicle or 200 *μ*M TMZ for 72 h. Whole-cell lysates from the viable cells were analyzed for luciferase activity. The relative expression in luciferase activities are presented as the mean±S.D. The data represent triplicates from four independent experiments, *n*=12 (**a**). Shown is the AP-1 site within the 5′ regulatory region *Cx43* (*GJA1)* (**b**). GBM cell lines were transfected with a reporter vector containing AP-1 Tandem Repeats. The transfectants were treated with 200 *μ*M TMZ. After 72 h, whole-cell extracts were studied for luciferase levels. The results are presented as mean±S.D. of triplicates in four independent experiments, *n*=12 (**c**). *P<0.05 *versus* vehicle

Figures 1 and 2 supported the involvement of Cx43 in TMZ resistance. We asked if this occurs by induced *Cx43*. We used a reporter gene system with four different fragments of the 5′ regulatory region of *Cx43*. U87 and T98G were transfected with the reporter gene vectors containing 250, 350, 400 and 1400 bp upstream of the translational start site.^21^ Controls were transfected with vector alone. After 24 h, the transfectants were treated with 200 *μ*M TMZ or vehicle. At 72 h, whole-cell extracts from the viable cells were analyzed for luciferase activity. The results, presented fold change over control, indicated a significant (*P*<0.05) increase in luciferase in the TMZ-resistant cells as compared with vehicle treatment (Figure 3a). As each fragment contained the first 250 bp of the *Cx43* gene, we concluded that this fragment was important for the activation of the reporter gene during TMZ treatment.

### TMZ activated AP-1

The 250-bp fragment within the 5′ regulatory region of *GJA1 (Cx43)* was identified as the active region in the TMZ-resistant cell lines (Figure 3a). To understand this further, we scanned the region for transcriptional factor binding site. We selected a potential AP-1-binding site (Figure 3b) because this site has been shown to induce *Cx43* in other tissues.^22^ We transfected the GBM cells with a reporter gene that was under the control of six AP-1 tandem repeats and then treated the transfectants with 200 *μ*M TMZ. At 24, 48 and 72 h, we analyzed cell extracts for luciferase activity. In both cell lines, luciferase levels were significantly (*P*<0.05) increased at 48 h, whereas this consistency was not seen at the 24-h time point (Figure 3c). The results indicated that TMZ treatment led to the activation of AP-1 in the GBM cells and this occurred after 48 h TMZ treatment. Based on these results, we proposed that AP-1 was activated about 24 h before TMZ resistance.^20^

### Interaction of AP-1 with endogenous *GJA1 (Cx43)*

AP-1 seemed to have a role in *Cx43* expression (Figure 3). As we did not mutate the AP-1-interacting sites, the studies could not definitively state that this region was responsible for the increase in *Cx43* transcription. We however used different methods to link the pathways in the activation of AP-1 in TMZ-resistant GBM cells.

**Figure 4:**
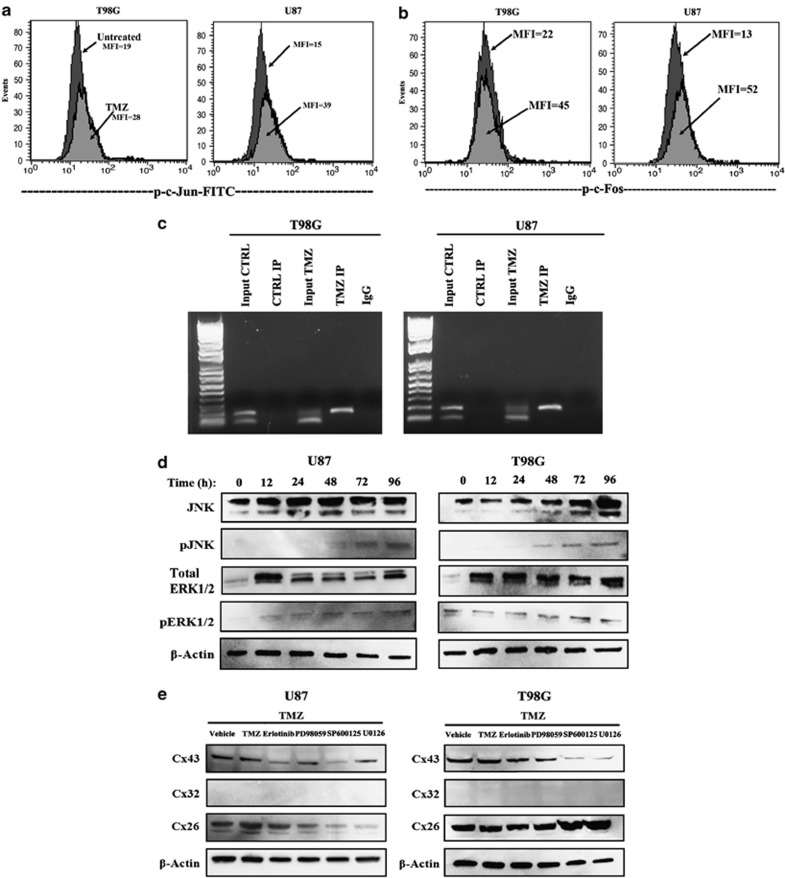
EGFR-mediated activation of AP-1 in TMZ-treated GBM cells. (**b**) U87 and T98G cells were treated with 200 *μ*M TMZ. After 72 h, the viable cells were analyzed by flow cytometry for phospho (p)-c-Jun (**a**) and p-c-Fos activation. The dark histograms represent untreated cells and the gray histograms, TMZ treatment. The MFI of each histogram is depicted with an arrow. (**c**) ChIP analyses were performed for AP-1 binding to endogenous *Cx43* using TMZ-resistant GBM cells. The complex was precipitated with anti-c-Jun. Shown are the PCR of the precipitated gDNA with primers spanning the AP-1 site. (**d**) Western blots were performed for the upstream activators of AP-1 activation, pERK and p-JNK using whole-cell extracts from untreated (Time 0) and timeline treatment of U87 and T98G with 200 *μ*M TMZ. The timeline studies occurred at 12 h intervals up to 96 h. (**e**) GBM cells were pretreated different pharmacological agents along the EGFR signaling pathway. After 24 h, the cells were washed and then exposed to 200 *μ*M TMZ for 72 h. Whole-cell extracts were analyzed by western blots for Cx26, Cx32 and Cx43

AP-1 is a heterodimeric transcription factor composed of c-Fos and c-Jun proteins. In addition, c-Jun members can also homodimerize.^23^ We therefore asked which subunits were activated in the TMZ-treated U87 and T98G cells. We treated the cells for 72 h with 200 *μ*M TMZ and then performed intracellular labeling for phospho-c-Jun and -Fos. Flow cytometric analyses indicated increased phospho-c-Jun in both cell lines and minimum phospho-c-Fos (Figures 4a and b, Supplementary Figure S2).

We next asked whether AP-1 interacted with endogenous *GJA1 (Cx43)* in TMZ-resistant GBM using chromatin immunoprecipitation (ChIP) assay with anti-c-Jun and PCR with primers spanning the AP-1 site. Analyses of the PCR product on agarose gel electrophoresis showed a bright band for TMZ-resistant GBMs. This indicated that AP-1 interacted with the *Cx43* gene in the TMZ-resistant GBM cells (Figure 4c).

### ERK and JNK in AP-1 activation

The results shown thus far indicated that AP-1 was important for the expression of *Cx43* in the TMZ-resistant GBM cells. We next asked whether the activation of AP-1 was because of upstream activation of JNK and ERK1/2. These kinases are downstream of EGFR signaling, which is commonly associated with GBM pathology.^24^ Thus, auto-transphosphorylation of EGFR could activate AP-1 via ERK1/2 and JNK. We performed western blot for total and phospho-ERK and -JNK in untreated (time 0) and TMZ-treated GBM cells. Specific antibodies were used to detect unphosphorylated and the phosphorylated forms (total). The results showed time-dependent increases in phospho-ERK and -JNK (Figure 4d and Supplementary Figure S2).

### EGFR1-JNK-ERK-AP-1 axis in *Cx43* expression

Studies were conducted to determine whether JNK-ERK-AP-1 axis (Figure 4d) was involved in *Cx* expression in the TMZ-resistant GBMs. Specific pharmacological inhibitors blocked JNK, ERK1/2 and EGFR activation. The activation of EGFR kinase was inhibited by pretreating GBM cells with Erlotinib for 24 h. Similarly, the cells were pretreated with PD98059 to block ERK1, SP600125 to inhibit JNK and U0126 to block the effect of ERK1/2. The cells were treated with 200 *μ*M TMZ for 72 h that were the parameters of resistance. Whole-cell extracts were analyzed by western blot for Cx43, −32 and −26. If the inhibitors sensitized the GBMs to TMZ this would reduce the number of GBMs. As the housekeeping *β*-actin was reduced, we normalized each lane of Cx to *β*-actin (Figure 4e).

The lanes labeled as vehicle represent the control with the inhibitors and TMZ treatment. As expected, the bands were bright as the GBMs retained resistance (Figure 1b). In contrast, cells treated with the kinase inhibitors resulted in decreased Cx43 (Figure 4e and Supplementary Figure S2).

### Increased activation of EGFR in TMZ-resistant GBM cells

**Figure 5:**
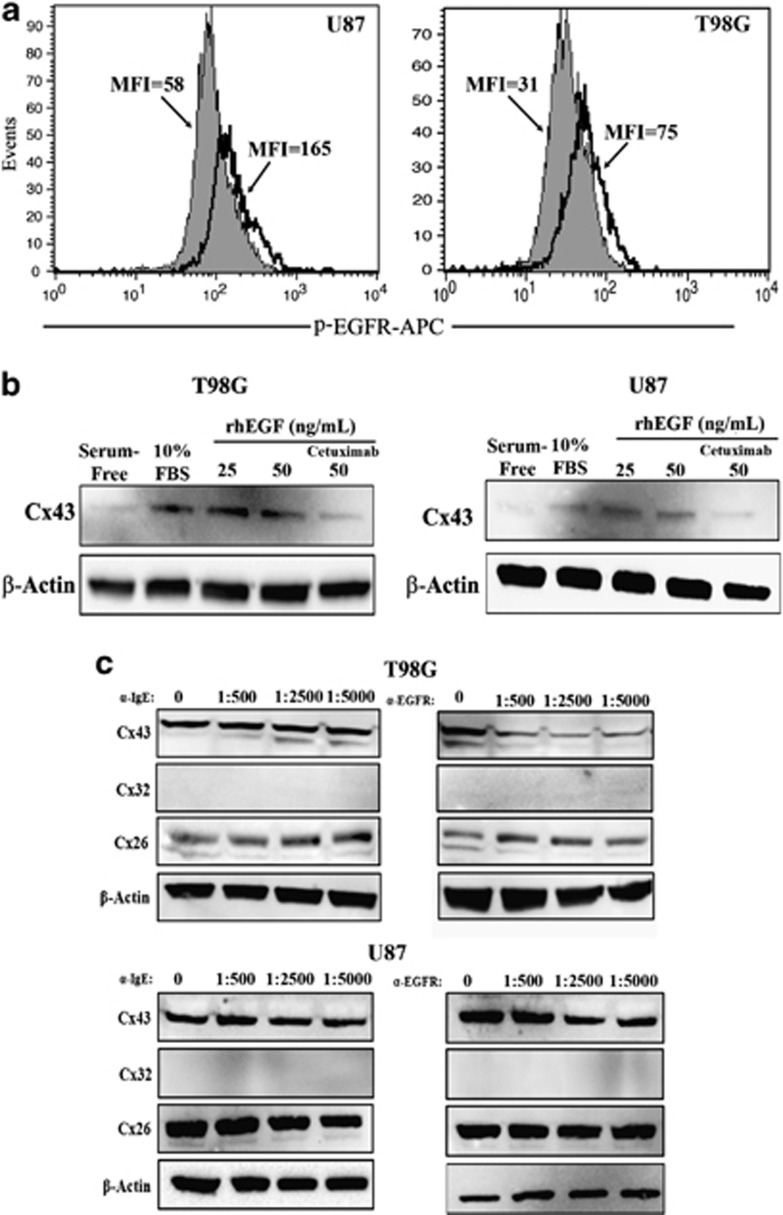
Effects of EGFR signaling on Cx43 expression. (**a**) Flow cytometry was performed for cell surface expression for EGFR with untreated and TMZ-resistant GBM cells. Resistance was established with 200 *μ*M TMZ for 72 h. The solid histogram represents untreated cells and the open histogram represents treated cells. The MFI of each histogram is shown with an arrow. (**b**) GBM cells were treated in sera-free media with 25 and 50 ng/ml of rhEGF. In parallel, the 50 ng/ml point also contained Cetuximab at 1/500 dilution. In parallel, the cells were stimulated with 10% FBS alone and with serum-free medium alone. At 24 h, whole-cell lysates were analyzed by western blots for Cx43. (**c**) TMZ-resistant U87 and T98G cells were established with 200 *μ*M TMZ, in the presence or absence of Cetuximab (*α*-EGFR) or Omalizumab (*α*-IGE). Each antibody was used at the following final dilutions: 1 : 500, 1 : 2500 and 1 : 5000. After 72 h, whole-cell lysates were analyzed by western blot for Cx26, Cx32 and Cx43

EGFR signaling has been associated to *Cx43* expression.^25^ As Cx43 was increased in the TMZ-resistant GBMs (Figure 1), we asked whether this was caused by EGFR activation as a large number of GBM patients showed increased/activated EGFR.^26^ GBMs were treated with 200 *μ*M TMZ for 72 h or vehicle (untreated) and then analyzed for surface EGFR by flow cytometry. Phosphorylated EGFR was increased by two- to three-fold in the TMZ-resistant cells (Figure 5a, open histogram for resistant cells).

The studies strongly suggested that activated EGFR was involved in *Cx43* expression (Figure 4e). We therefore performed cause–effect analyses by activating EGFR with exogenous rhEGF in sera-free media containing 200 *μ*M TMZ and exogenous rhEGF (IC_50_=50 ng/ml). After 72 h, whole-cell extracts were studied for Cx43 by western blot. The results showed significant (*P*<0.05) increase in Cx43 protein in the presence of rhEGFR as compared with untreated/sera-free GBM cells (Figure 5b, Supplementary Figure S3). The specificity of EGF was tested by blocking the effect of EGFR with FDA-approved anti-EGFR chimeric monoclonal antibody (Cetuximab) (Figure 5b, right lanes).

The use of Cetuximab in sera-free media indicated that EGFR is involved in *Cx43* expression (Figure 5b). We therefore repeated the studies in which TMZ-resistant GBM cells were cultured in sera-containing media. The specificity for EGFR was studied with different amounts of Cetuximab. We did not use Erlotinib to block the kinase activity of EGFR but selected Cetuximab because it competed with EGF. The EGF can be present in the sera or produced by the resistant GBM cells. Thus, Cetuximab will prevent autocrine and paracrine stimulation of EGFR.

As expected, in the absence of Cetuximab (experimental point 0), the bands for Cx43 was bright, which is consistent for the TMZ-resistant cells (Figures 1b and 4e). However, in the presence of Cetuximab, the bands for Cx43 were decreased as compared with parallel cultures without Cetuximab (Figure 5c, 0 *α*-EGFR; Supplementary Figure S3). Interestingly, the decrease in the bands also correlated with the disappearance of the lower bands, which are generally indicative of phosphorylated Cx43.^27^ The results with Cetuximab were specific because similar studies with an unrelated chimeric monoclonal anti-IgE (FDA approved, Omalizumab) did not change the bands for Cx43 (Figure 5c, left panels and Supplementary Figure S3). Together, the studies showed a role for EGFR signaling in the expression of *Cx43* in the TMZ-resistant GBM cells.

### Dye transfer between GBM cells

**Figure 6:**
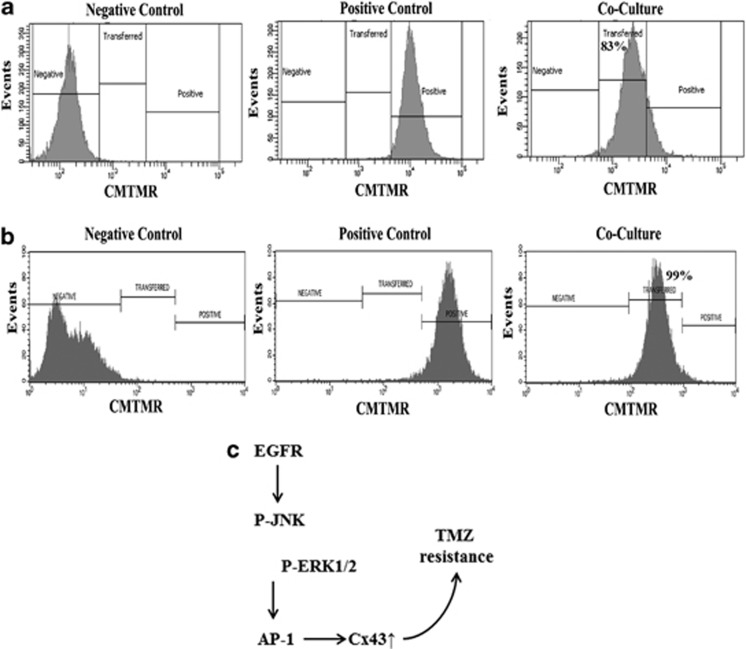
GBM cells form functional gap junctions. (**a**) Ten^5^ unlabeled U87 or T98G cells were cocultured for 72 h with ten^5^ CMTMR-labeled (Cell Tracker Orange) cells (center). Dye transfer was evaluated by flow cytometry. (**b**) A diagram shows TMZ-resistant GBM cells with activated EGFR, which phosphorylated JNK and ERK1/2 to activate AP-1 for *Cx43* expression

As the resistant GBM cells expressed *Cx43*, we asked whether the Cx43 was functional with respect to the formation of GJIC between the resistance GBM cells. Resistant GBM cells were established by treating the cells with 200 *μ*M TMZ for 72 h as described.^20^ The resistant GBM cells were cocultured at 1:1 ratio of unlabeled and CMTMR (Texas Red)-labeled. Negative controls are represented as unlabeled GBM cells and positive controls as labeled GBM cells alone. After 72 h, dye transfer was assessed by fluorescence microscopy and flow cytometry. As compared with the initial 50% labeled cells by 72 h, >90% of the cells contained the CMTMR dye. This indicated that the dye was transferred from one cell to the other (Figure 6, Supplementary Figure S4). Parallel studies with 1-octanol prevented the dye transfer, indicating specificity of GJIC. In four different experiments, the transfer of dye was >80% efficiency between the TMZ-resistant GBM cells. Representative transfer shows 83.2% for U87 and 98.8% for T98G (Figure 6a).

## Discussion

This study reported on increased expression of *Cx43* in TMZ resistance GBM cell lines and low-passage cells (Figures 1 and 2). TMZ induced *Cx43* transcription by an increase in EGFR that activated MAPK-AP-1 (Figures 3). The findings were supported by the data in TCGA (Figure 1e). We have analyzed the chemoresistance to TMZ in long-term- (>3 months) and short-term (72 h)-treated GBM cells (unpublished data). The treatment led to ∼40% cell death in both the short- and long-term exposure to TMZ; hence the 72-h time period was selected to induce TMZ resistance.

The ChIP assay combined with pharmacological inhibitors supported a role for AP-1 in *Cx43* induction. The present report did not use the reporter gene constructs with mutant AP-1 site. Such studies are planned to fully understand how increased Cx43 causes chemoresistance in GBM cells. TMZ-mediated increase in *Cx43* transcription in the GBM cells would allow the formation of GJIC for exchange of molecules across gap junction (Figures 6a and b). We reported on the transfer of miRNAs from chemoresistant GBM cells through exosomes.^20^ As miRNAs can be passed from one cell to another through GJIC,^14^ our findings could be another method by which resistant GBM cells survive TMZ treatment.

The resistance of GBM to TMZ with tumor relapse within months is important to understand the mechanism of resistance.^28^ This study identified potentially new methods to combine with current treatments for GBM. We showed how TMZ could cause GJIC for the passage of small molecules (<2 kDa) such as miRNA.^29^ In the CNS, astrocytes abundantly express gap junctions for cellular maintenance and regulation.^30^ Cx43 is the main contributor of GJIC formation in astrocytes.^16^ Astrocytomas commonly overexpress the same gap junction proteins as shown here for GBM, which is World Health Organization grade IV astrocytomas.^31^ The identification of Cx43 as key in the resistance of GBM to TMZ is in line with the argument that this could be important for the migration of GBM.^18^

The systematic approach used in this study showed a role for Cx43 in the resistance of GBM to TMZ. The significance of our findings in future studies was demonstrated in our final set of studies in which we showed functional GJIC between GBM cells by fluorescent dye transfer (Figure 6). The importance of the gap junctional protein in TMZ resistance was underscored in studies in which we knocked down *GJA1* and demonstrated an increase in cell death to TMZ (Figure 2). Together, these experiments supported a role for Cx43 as a protector against TMZ treatment.

We analyzed the region within the *GJA1* that showed sensitivity to TMZ using a reporter gene system.^27^ These studies indicated that TMZ can activate the region of *GJA1* close to the transcriptional start site (Figure 3). The region contained an AP-1 site (+44/+50), which was shown to be functional with regard to *Cx43* expression.^22^ Indeed, we showed that the AP-1 subunit, c-Jun, was phosphorylated after TMZ exposure (Figure 4d). This correlated with the ability of AP-1 to bind endogenous *GJA1*, as indicated by the ChIP assay (Figure 4c).

Overall, the findings showed a role for AP-1 in the induction of *MDR1* in resistant GBM cells. Thus, it is expected that AP-1 transcription should be increased before full resistance, which occurs with 72 h TMZ treatment. Indeed, timeline studies using a reporter gene under the control of AP-1 repeats showed an increase in AP-1 activity at 48 h of TMZ treatment (Figure 3c). This correlated with an increase in the phosphorylated form of AP-1 subunit at 72 h (Figure 4a). The phosphorylation of c-Jun is downstream of activated ERK1/2 and JNK (Figure 4d). These activated proteins were involved in *Cx43* expression (Figure 4e). As ERK1/2 and JNK can be activated by the EGFR, we focused on the involvement of EGFR on TMZ resistance of GBM cells. This data indicated an increase of functional EGFR expression, resulting in an increase in *Cx43* expression (Figure 5). These findings suggested that EGFR signaling could be important even in cases where the receptor was not shown to be motivated. As a monoclonal antibody to EGFR was approved as Cetuximab by the FDA, the findings show promise for the treatment of GBM. These findings underscore the importance of taking another review on the pathogenesis of EGFR in the biology of GBM. In our ongoing studies, we have performed *in vivo* studies in which we were able to reverse TMZ resistance with an inhibitor of EGFR kinase. The findings will be the subject of a separate paper.

Data derived from >500 GBM tissues in TCGA supported the *in vitro* findings on increased Cx43 GBM cells as compared with Cx26 and Cx32 (Figure 1e). The findings were validated with two low-passage cell lines derived from an untreated patient (BT145) and TMZ-resistant (BT164) GBM patient. More importantly, the two low-passage cell lines supported the expression of Cx43 on TMZ-resistant cells. As BT145 was never treated, it is assumed that this cell line was TMZ sensitive. Although the mRNA for the three Cxs were increased in the resistant line, only Cx43 protein was increased in the resistant BT164 cells and the resistant established GBM cell lines (Figure 1).

Figure 6c summarizes the main findings in this study. We showed EGFR signaling as essential for the increase in Cx43 in the TMZ-resistant GBM cells. The resistant GBM cells expressed higher level of EGFR (Figure 5a). However, it was unclear whether the increase was caused by TMZ. This question requires a separate study to understand how TMZ induces EGFR and how to discriminate between unphosphorylated and phosphorylated EGFR. As we demonstrated GJIC in the chemoresistant GBM cells, the findings indicated that EGFR signaling could be significant for intercellular communication and might explain how the resistance of one cell may be spread to other similar cells. The initial studies with immortalized cell lines were supported with other sources of GBM: array databases with patients' tissues and with low-passaged cell lines from GBM. The use of pharmacological methods to decrease Cx43 was encouraging, as this strategy could be explored for new methods to treat GBMs. Direct targeting of Cx43 might be toxic as neural cells express high level of Cx43.^32^ On the other hand, this study opens the ‘door' to research that could lead to the identification of molecules that are exchanged between the resistant GBM cells. Their identity could be new drug targets. We showed that mesenchymal stem cells can be used to deliver anti-miRNA to resistant GBM cells.^20^ Similar methods could be used once future studies identified the molecules that are exchanged through GJIC. The proposed method with stem cells have been used to transfer prodrug to brain tumors.^33^

## Materials and Methods

### Cell lines

U87 and T98G World Health Organization grade IV GBM cells were purchased from American Type Culture Collection (Manassas, VA, USA) and then expanded as per manufacturer's instructions.

BT145 (primary GBM) and BT164 (recurrent GBM) glioma cell lines were derived from surgical resection material acquired from patients undergoing surgery at the Brigham and Women's Hospital on an Institutional Review Board approved protocol as part of the DF/BWCC Living Tissue Bank. Briefly, tumor resection samples were mechanically dissociated, and tumorspheres were established and propagated in Human NeuroCult NS-A Basal media (StemCell Technologies, Vancouver, BC, Canada) supplemented with EGF, FGF*β* and heparin sulfate.

### Reagents and antibodies

All tissue culture media were purchased from Gibco (Grand Island, NY, USA), and fetal calf serum was purchased from Hyclone Laboratories (Logan, UT, USA). Recombinant human EGF was obtained from R&D Systems (Minneapolis, MN, USA). For fluorescent imaging, CellTracker Orange CMTMR [5-(and-6)-(4-chloromethyl) benzoyl)amino)tetramethylrhodamine] was purchased from Invitrogen (Carlsbad, CA, USA).

Mouse anti-Cx43, anti-Cx32 and anti-Cx26 were purchased from Invitrogen and, mouse anti-*β*-actin mAb, HRP-anti-rabbit and HRP-anti-mouse IgG from Cell Signaling Technology (Billerica, CA, USA). EGFR blocking humanized antibody (Cetuximab) and humanized IgE (Omalizumab) were kind gifts from Roche (Nutley, NJ, USA).

EGFR signaling inhibition was achieved using the following inhibitors: Erlotinib (10 *μ*M, IC_50_=5–20 *μ*M), SP600125 (25 *μ*M, IC_50_=10–50 *μ*M), PD98059 (5 μM, IC_50_=5–10 *μ*M) and U0126 70 *μ*M, IC_50_=50–70 *μ*M). SP600125, PD98059 and U0126 were purchased from Sigma-Aldrich (St. Louis, MO, USA) and Erlotinib was purchased from Santa Cruz Biotechnology (Santa Cruz, CA, USA).

### Vectors

The *GJA1* (*Cx43*) reporter gene (luciferase) contained inserts within the 5′ regulatory region. The reporter gene vectors were kindly provided by Dr. M Kandouz, Wayne State University School of Medicine (Detroit, MI, USA). The vectors were previously described.^21^ The pGL3 luciferase backbone vector (Promega, Madison, WI, USA) served as vector control. AP-1 activity was evaluated with pGL4.44[*luc2P*/AP-1 RE/Hygro, which is a 6x Tandem Repeat Element of AP-1, upstream of luciferase (Promega).

### Dye transfer

U87 or T98G cells (5 × 10^5^) were seeded in six-well plates and then incubated with 2.5 *μ*M of prewarmed CellTracker Orange CMTMR [5-(and-6)-(((4-chloromethyl) benzoyl)amino) tetramethylrhodamine] (Life Technologies - Molecular Probes, Grand Island, NY, USA). After the CMTMR enters the cells, it reacts with the intracellular components and cannot exit the cells. Thus to ensure specificity, GJIC studies are done in the presence of an inhibitor, 1-octanol. The cells were incubated for 45 min at 37 °C in a CO_2_ incubator. After this, the media were removed and the cells were washed three times with PBS. Fresh culture media were added to the washed cells. The plates were incubated for 6 h and then subjected to a second wash with PBS. After this, the labeled cells were cocultured with 5 × 10^5^ unlabeled cells. After 72 h, the cells were trypsinized, centrifuged at 500 × *g*, resuspended in 0.5 ml of PBS and then immediately evaluated by flow cytometry for dye transfer.

### Real-time RT-PCR

RNA was extracted with Trizol reagent (Invitrogen). Reverse transcription was performed using the High Capacity cDNA Reverse Transcription Kit (Life Technologies - Applied Biosystems, Grand Island, NY, USA) in accordance with the manufacturer's recommendation. Real-time PCR was performed with 200 ng cDNA on a 7300 Real-Time PCR System (Applied Biosystems). The initial incubation was at 50 °C for 2 min followed by 95 °C for 10 min. After this, the cycling conditions were as follows: 95 °C for 15 s and 60 °C for 60 s, for 40 cycles. Primer sequences used in the PCR were: Cx26: (F) 5′-AGCTCTGCTCCCCTAAAG-3′ (R) 5′-TGTGTCCTCTGTGGAACC-3′ Cx32: (F) 5′-GGCATTCTACTGCCATTG-3′, (R) 5′-TGGGGTGGAAACTAGGAT-3′ Cx43: (F) 5′-TCGGGTTAAGGGAAAGAG-3′, (R) 5′-GCTCACTTGCTTGCTTGT-3′ *β*-actin: (F) 5′-TGCCCTGAGGCACTCTTC-3′ and (R) 5′-GTGCCACCAGGGCAGTGATCT-3′. Primers were purchased from Sigma (St. Louis, MO, USA). The relative expression was calculated using 2(−Delta Delta C(T)), as previously described.^14^

### Western blot

GBM cells were treated with 200 *μ*M TMZ or with vehicle. After 72 h, whole-cell extracts were isolated with M-PER Mammalian Protein Extraction Reagent (Thermo Scientific, Rockford, IL, USA). Cell extracts (3–7 *μ*g) were analyzed by western blots, as previously described on 12% SDS-PAGE gels (Bio-Rad, Hercules, CA, USA).^27^ Proteins were transferred to PVDF membranes (PerkinElmer, Boston, MA, USA). The membranes were incubated overnight with primary antibodies at final dilutions ranging between 1/500 and 1/1000. Primary antibodies were detected by incubating for 2 h with HRP-conjugated IgG at 1/2000 final dilution. HRP activity was assayed by chemiluminescence using SuperSignal West Femto Maximum Sensitivity Substrate (Thermo Scientific). Membranes were stripped with Restore Stripping Buffer (Thermo Scientific) and then reprobed with other antibodies.

### Flow cytometry

The cell surface expression for EGFR was analyzed by flow cytometry with 1/100 final dilution of anti-EGFR (Cetuximab with human Fc). This was followed by incubation with 1/500 mouse anti-human IgG (bridging antibody). A third incubation used 1/1000 FITC-conjugated goat-anti-mouse IgG. The fluorescence intensity was determined with a FACS Calibur (BD Biosciences, San Jose, CA, USA).

### Cell death analyses

Lactate dehydrogenase (LDH) release assays (Promega) studied cell death. The LDH assay was based on the disruption of the cell membrane. The LDH assay was assessed at 72 h after TMZ treatment.^34^ We measured the conversion of Formazan (Red) using a Victor3V Multilabel Plate Reader (PerkinElmer; Waltham, MA, USA) with an emission filter of 490 nm. The results were normalized to complete cell lysis. This was aided by a lysis buffer included in the LDH assay kit.

### Analyses of the 5′ regulatory region of *GJA1*

The minimal promoter of the *GJA1* gene was identified on the basis of the information in the NCBI database (NC_000006.11). This region was identified as −250 bp, relative to the transcriptional initiation site, and was also supported by luciferase activity (see Results section). The 250-bp region was analyzed for potential transcriptional binding regions using Genematix platform (Munich, Germany).

### ChIP assay

ChIP analysis for AP-1-interacting site on the *GJA1* gene was performed using the ChIP-it Express Enzymatic Kit (Active Motif, Carlsbad, CA, USA), as described.^35, 36^ A ChIP-validated anti-c-Jun antibody was also purchased from Active Motif. GJA1 isolation was confirmed by qPCR. Samples were isolated during the exponential phase of synthesis as determined by qPCR and run on 1% agarose gel. Although two bands were amplified from genomic input DNA, only the upper band (200 bp) was isolated by ChIP. The upper band was confirmed to be GJA1 PCR.

### Transfection and reporter gene assay

U87 and T98G cells were transfected with the *GJA1* reporter gene constructs, using Effectene Transfection Reagent (Qiagen, Valencia, CA, USA). After 72 h, protein lysates were obtained using M-PER Mammalian Protein Extraction Reagent (Thermo Scientific), and quantified for protein concentration (Bio-Rad). Luciferase activity was calculated as per total protein and then presented as relative light units. The relative light units was normalized to untreated lysates.

### Statistical analysis

Data were analyzed using the Student's *t*-test for two comparable groups (control/experimental). A *P*-value <0.05 was considered significant.

## Abbreviations

Cx: connexin
Cx43: connexin 43
EGF: epidermal growth factor
EGFR: epidermal growth factor receptor
GBM: glioblastoma
*Gja1*: connexin 43–deficient mice
GJIC: gap junctional intercellular communication
TCGA: The Cancer Genome Atlas
TMZ: temozolomide
