# Supplementary material for: Temozolomide resistance in glioblastoma cells occurs partly through epidermal growth factor receptor-mediated induction of connexin 43
Source: Cell Death Dis. 2014 Mar 27;5(3):e1145–. doi: 10.1038/cddis.2014.111 (PMC3973225; doi:10.1038/cddis.2014.111)
Supplement: Supplementary Information [file cddis2014111x1.doc]

**SUPPLEMENTAL INFORMATION**

**
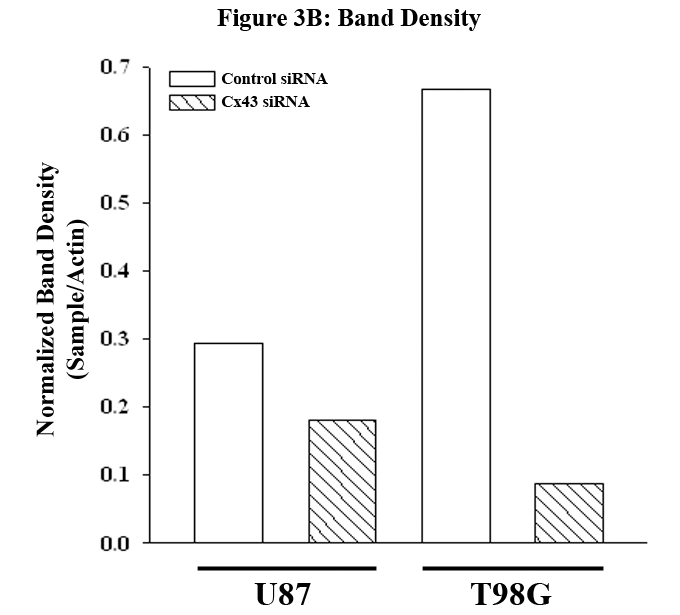
**

**Figure S1:** Band Density analyses for the western blot in Figure 2B were performed with UN-SCAN-IT software.

**
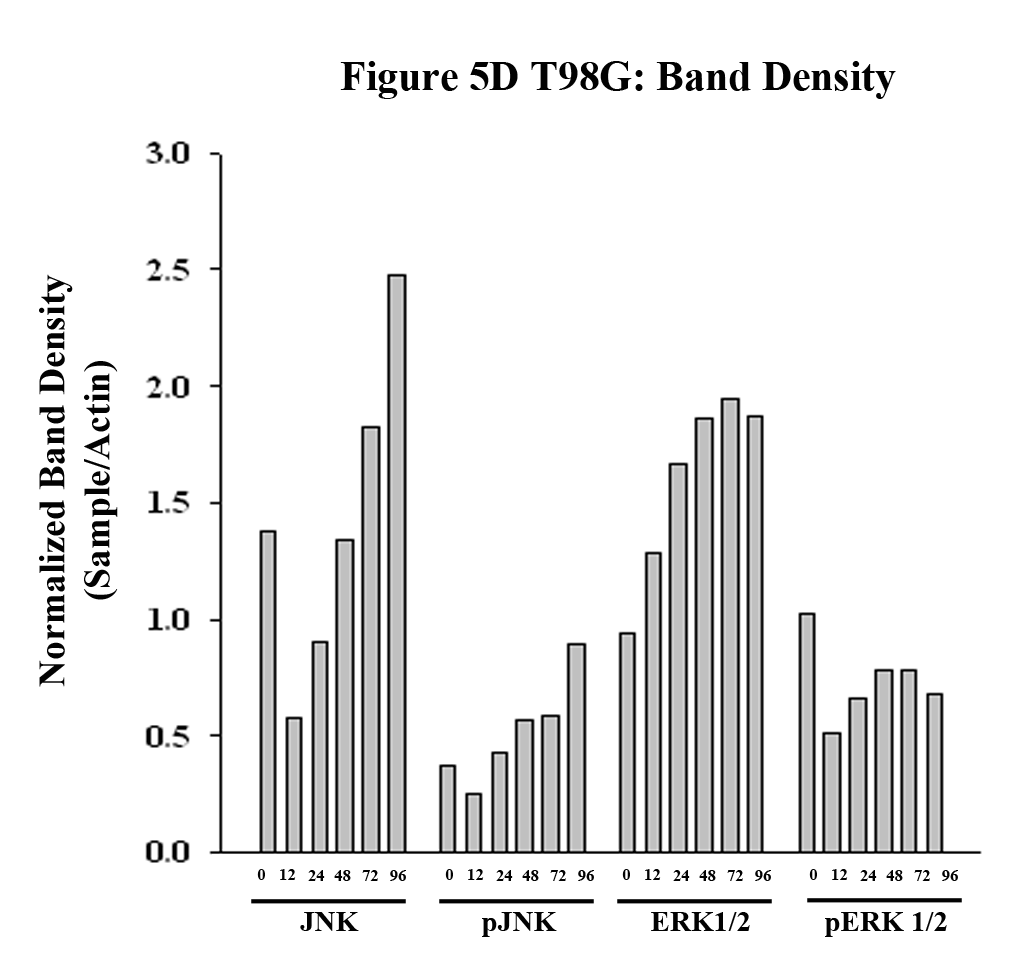

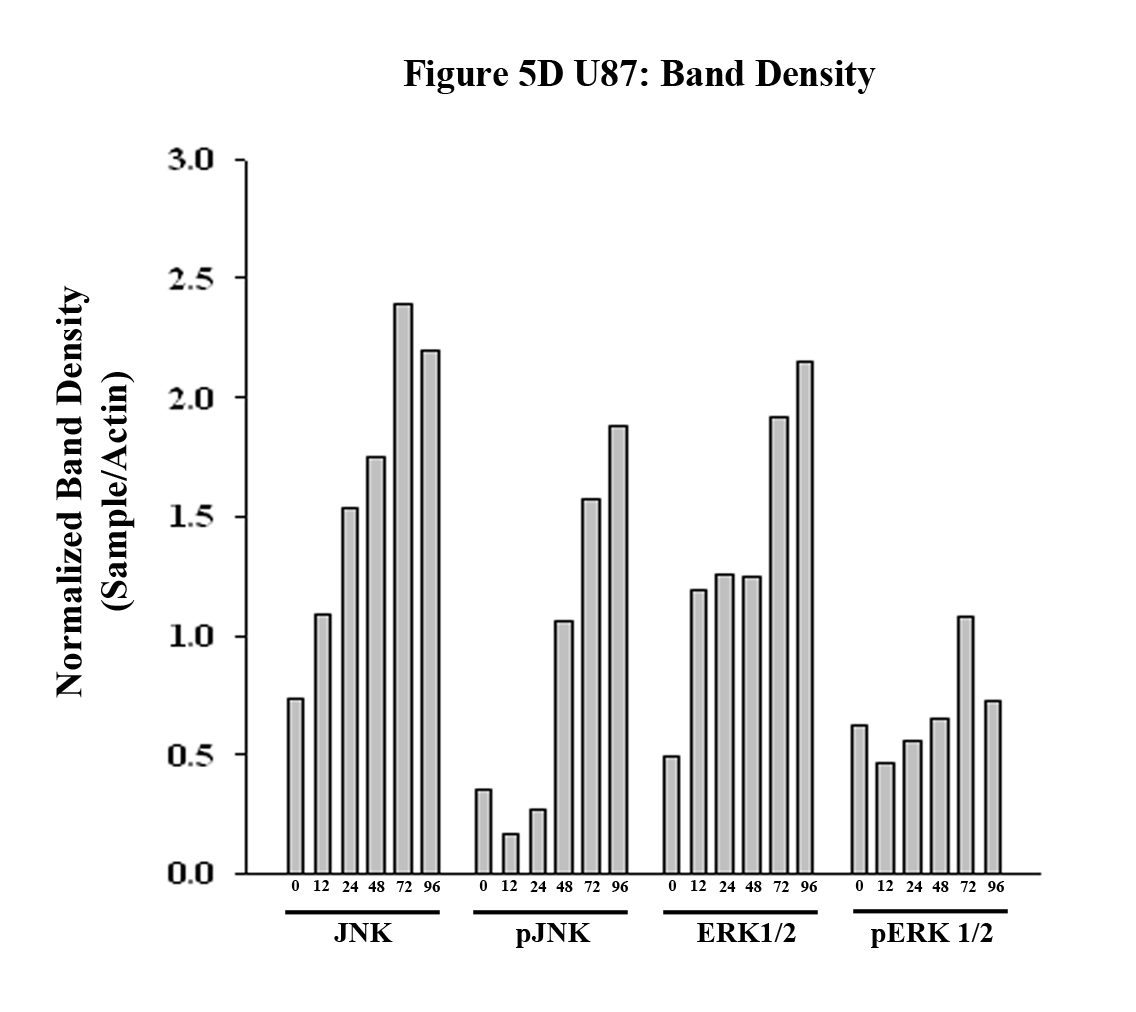

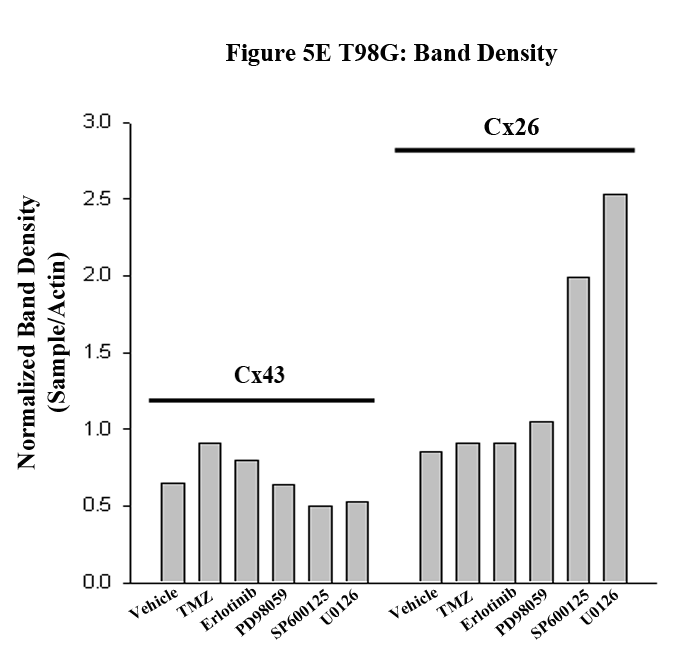

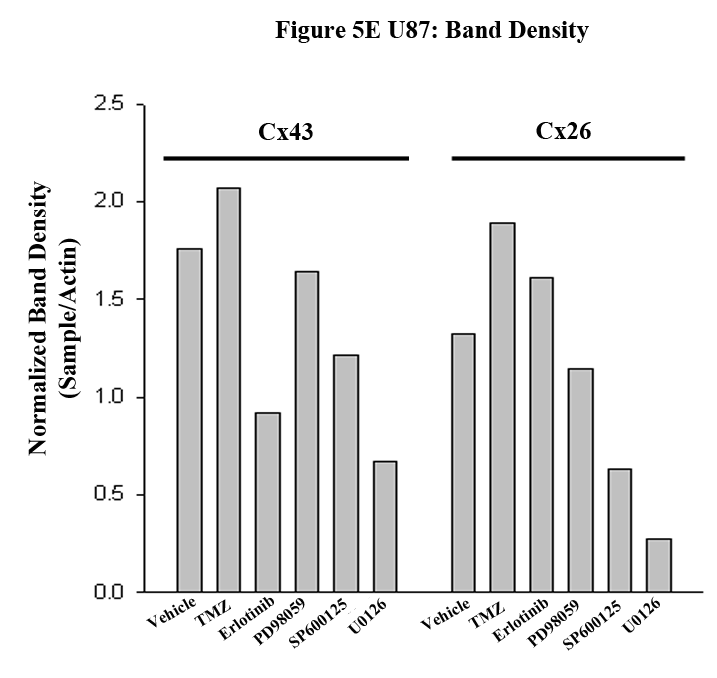
**

**Figure S2:** Band density analyses for the western blots in Figure 4 were performed with UN-SCAN-IT software.

**
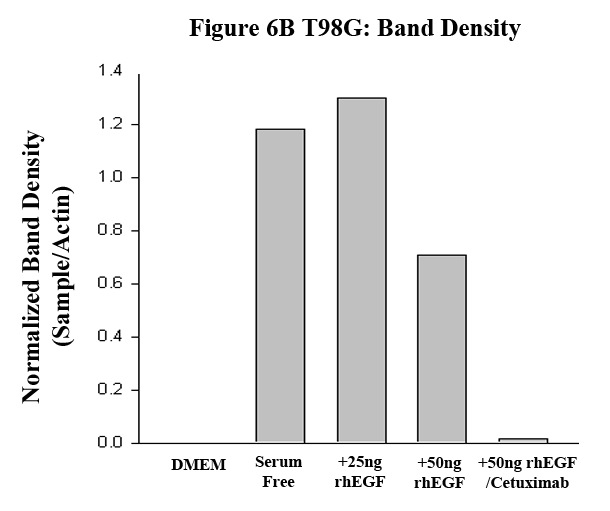

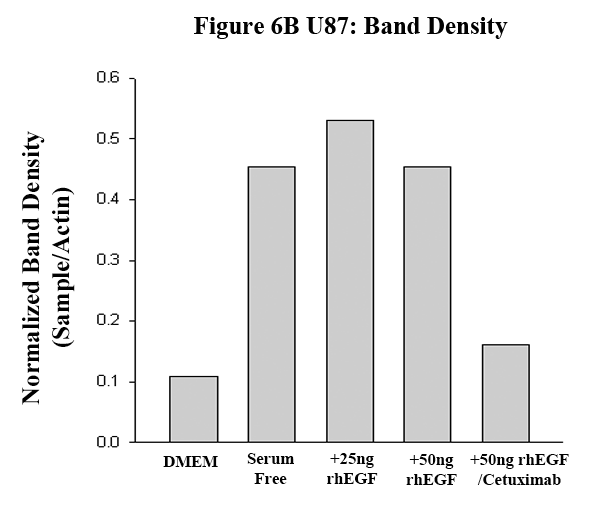
**

**
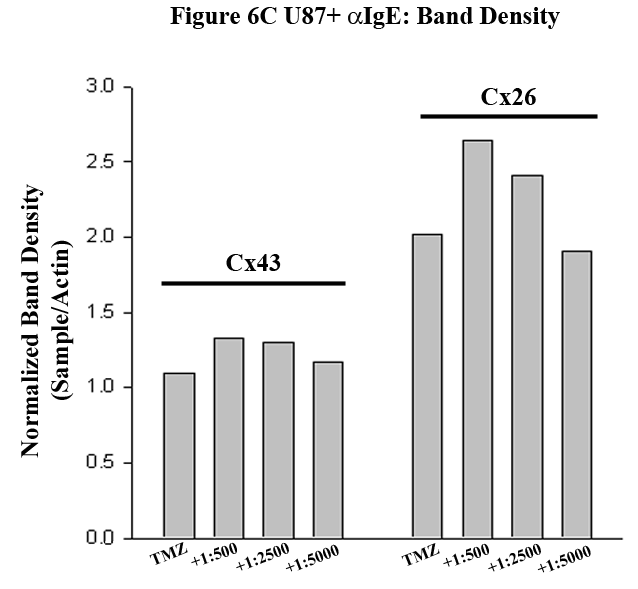

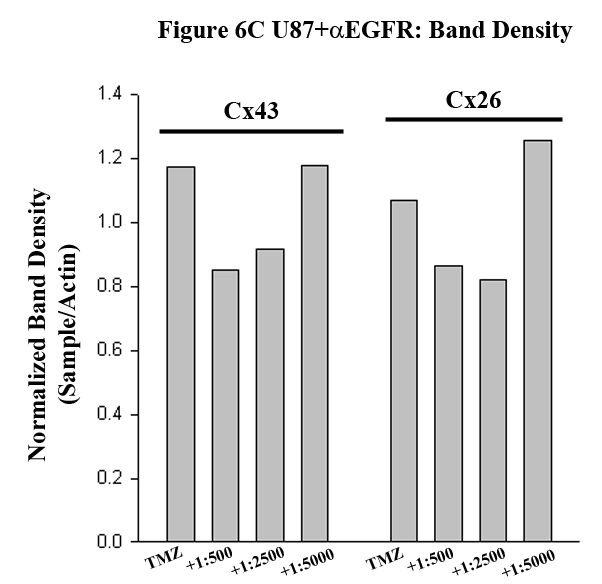
**

**
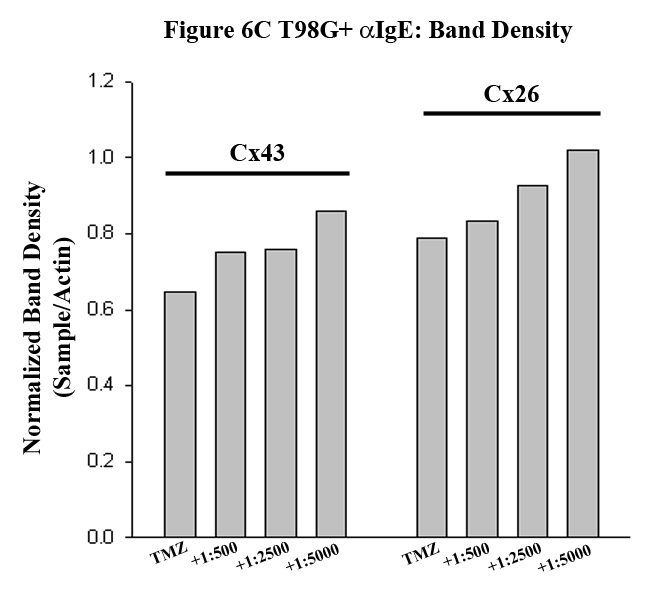

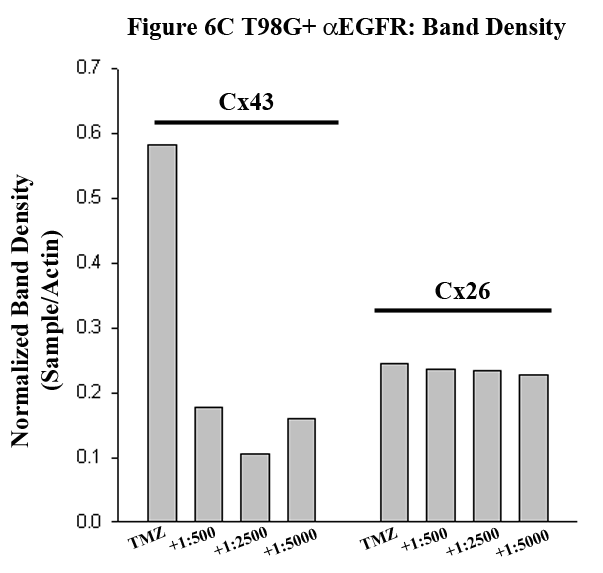
**

**Figure S3:** Band density analyses for the western blots in Figure 5 using UN-SCAN-IT software.


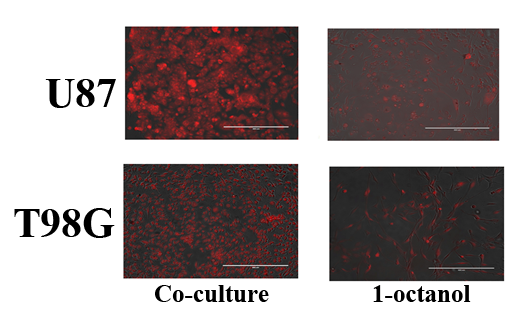


**Figure S4: Fluorescent dye transfer between resistant GBM cells.** Unlabeled TMZ-resistant U87 and T98G cells were co-cultured with equal amounts (10^5^ each) of CMTMR-labeled TMZ resistant U87 and T98G, respectively. After 72 h, the transfer of CMTMR dye was studied by fluorescent microscopy using the EVOS fl fluorescence imager (AMG Micro, Bothell, WA). The specificity of dye transfer was studied with 1-octanol as described [1, 2]. The results showed diffusion of the dye in >90% of all cells within the co-cultures, indicating that 40% was transferred. The addition of 1-octanol blocked the dye transfer.

References

1. Lim PK, Bliss SA, Patel SA, Taborga M, Dave MA, Gregory LA, et al. Gap Junction-Mediated Import of MicroRNA from Bone Marrow Stromal Cells Can Elicit Cell Cycle Quiescence in Breast Cancer Cells. *Cancer Res* 2011; **71**: 1550-60.

2. Park JH, Lee MY, Heo JS, Han HJ. A potential role of connexin 43 in epidermal growth factor-induced proliferation of mouse embryonic stem cells: Involvement of Ca2+/PKC, p44/42 and p38 MAPKs pathways. *Cell Prolif* 2008; **41**: 786-802.
